# Detection of Molecular Oxygen at Low Concentrations Using Quartz Enhanced Photoacoustic Spectroscopy

**DOI:** 10.3390/s100908466

**Published:** 2010-09-09

**Authors:** Andreas Pohlkötter, Michael Köhring, Ulrike Willer, Wolfgang Schade

**Affiliations:** 1 Clausthal University of Technology, Institute for Energy Research and Physical Technology, Energy-Campus, Am Stollen 19, 38640 Goslar, Germany; E-Mails: a.pohlkoetter@pe.tu-clausthal.de (A.P.); u.willer@pe.tu-clausthal.de (U.W.); 2 Fraunhofer Heinrich Hertz Institute, Energy-Campus, Am Stollen 19, 38640 Goslar, Germany; E-Mail: michael.koehring@hhi.fraunhofer.de (M.K.)

**Keywords:** Gas sensing, photoacoustic spectroscopy, QEPAS, oxygen sensing

## Abstract

Molecular oxygen is detected at low concentrations using photoacoustic spectroscopy despite its unfavorable photoacoustic properties. The system consists of a seed laser diode, a tapered amplifier and a quartz tuning fork based spectrophone, thus employing quartz enhanced photoacoustic spectroscopy (QEPAS). With this system a detection limit of 13 ppm is reached with a compact and long term stable setup. Further improvement of the detection limit is possible by adding suitable gases to the sample gas that promote the radiationless de-excitation of the oxygen molecules.

## 1. Introduction

An accurate determination of the concentration of molecular oxygen is important for the investigation of corrosion processes and in basic materials science research. Fields of application are also industrial process control and medical devices for oxygen monitoring.

The main advantages of laser based optical gas sensors are the selectivity and the low detection limits that can be reached, in combination with the capability of on-line and *in situ* measurements without the need for sample preparation.

The $X^{3}\Sigma_{g}^{-}=>b^{1}\Sigma_{g}^{+}$ absorption band around 762 nm is often used for absorption based optical sensing as it is the strongest optical transition of oxygen in the visible and infrared range. However, this optical transition is dipole forbidden, leading to a comparatively low maximum line strength of *S* = 8.5·10^−24^ cm^−1^/(molecule·cm^−2^) [1]. Different spectroscopic techniques have been applied to achieve a low detection limit even with this small line strength, including multi-path cells (35 ppm) [2] and cavity ring down spectroscopy, with less than 1 ppm detection limit [3,4]. However, despite their favorable detection limit, they pose some disadvantages: The setup is often difficult to adjust and bulky and the measurement volume is often large, *i.e.*, a large amount of gas is needed for a single measurement.

In this work, photoacoustic spectroscopy (PAS) is used for the sensitive detection of oxygen. For PAS a modulated laser irradiates a gas volume within a cell. If the wavelength of the laser coincides with an absorption line of the gas, the gas molecules are excited due to absorption of the light. Radiationless de-excitation leads to a heat induced pressure change within the gas volume. Because of the modulation of the exciting light, this pressure change is periodic, thus a sound wave is generated which can be recorded with a standard miniaturized microphone. For signal enhancement, the geometry of the gas cell is customary designed to be resonant for the generated acoustic wave. Although PAS is known as a cost effective, simple and sensitive spectroscopic method, few reports exist for sensitive oxygen detection [5]. This is due to the poor photoacoustic properties of oxygen. In this paper sensitive oxygen detection with quartz enhanced photoacoustic spectroscopy in combination with a powerful excitation source is demonstrated. A detection limit of 13 ppm is obtained.

## 2. Experimental

### 2.1. Experimental background

In general, a photoacoustic signal is generated by the heat induced pressure change due to radiationless transition of optically excited molecules. Since the exciting radiation is temporally modulated, the periodical pressure change will produce a sound wave that can be detected with a microphone. The photoacoustic signal *S* induced by the optical power *P* can be described by [6]:

(1) $$S∼\frac{\sigma \cdot N\cdot P\cdot Q}{A\cdot f}.$$

where *N* is the density of absorbing molecules with absorption cross section σ, *A* is the cross section of the acoustic resonator with resonance frequency *f* and *Q* is the quality factor of the resonant system.

**Figure 1.**
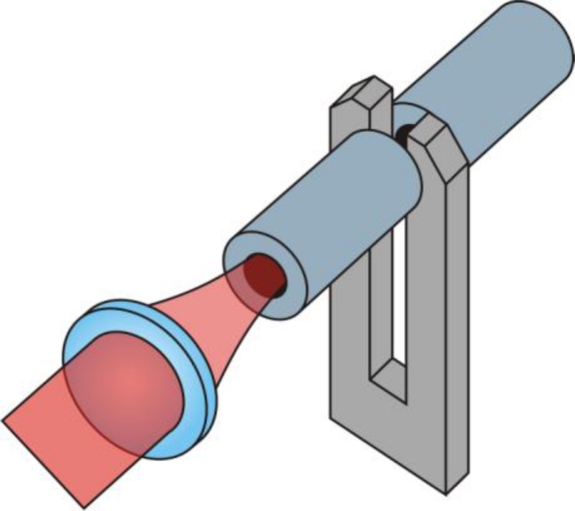
Illustration of a QEPAS spectrophone. The light is focused through the acoustic resonator tubes and the prongs of the tuning fork.

A recent development in photoacoustics is the so-called quartz enhanced photoacoustic spectroscopy (QEPAS) [6,7]. Instead of a microphone a quartz micro tuning fork (QTF) is used to detect the acoustic sound wave. To do so, the laser light is focused between the prongs of the micro tuning fork (Figure 1) which is driven into oscillation by a periodic pressure variation generated if absorbing molecules are present in the volume between the prongs. The amplitude of the oscillation is determined by measuring the piezo current that is generated due to deflection of the prongs of the quartz tuning fork. This device is insensitive to acoustic background noise because only an acoustic wave produced in between the prongs can give rise to a signal [7]. A commercially available QTF is used. The standard resonance frequency of approx. *f_0_* = 32 kHz for these devices also assures insensitivity to acoustic background noise since it decays with 1/*f*. The main difference between traditional PAS and QEPAS is thus the transducer used to convert the acoustic wave into an electric signal.

QEPAS is well known for being a simple to adjust and rugged spectroscopic technique without the need for long absorption path lengths. As the dimensions of the tuning fork are only 6 mm × 1.5 mm × 0.3 mm the photoacoustic cell can be reduced in size significantly compared to conventional photoacoustic spectroscopy. Therefore, the sample volume can be very small (*V* < 1 cm^3^), which is helpful for applications were only a small amount of gas is available.

The high Q-factor of the micro tuning fork (*Q* = 10^4^ – *Q* = 10^5^, depending on the gas pressure) results in a high signal enhancement and thus low detection limits. The signal can be further enhanced by an acoustic resonator consisting of two tubes attached in close vicinity to the tuning fork as depicted in Figure 1. Equation (1) is also valid for QEPAS if adequate parameters for *f* and *Q* are chosen: for the frequency *f* the resonant frequency *f_0_* of the tuning fork has to be inserted. The quality factor is not solely determined by the photoacoustic cell as in PAS but is influenced by the whole spectrophone, consisting of QTF and acoustic resonator.

Photoacoustic spectroscopy is rarely used for the detection of oxygen as the photoacoustic performance is known to be poor. This is a result of the molecular energy states of the oxygen molecule. Normally a rotation-vibration transition in the electronic ground state is used in photoacoustics. The $X^{3}\Sigma_{g}^{-}=>b^{1}\Sigma_{g}^{+}$ transition of the O_2_ molecule is a dipole forbidden electronic transition, with vibrational and rotational structure. The transition is nevertheless optically active due to its permanent magnetic dipole momentum in the triplet ground state. However, the transition probabilities for optical excitation are small. Since an electronic transition is included in the de-excitation process not only the collision induced vibration-translation energy transfer process (V-T process) can lead to a heating of the gas but also the electronic-translation process (E-T process) can occur during collisions of the excited oxygen with other molecules.

In this work the P7P7 rotational line within the first vibrational band of the $X^{3}\Sigma_{g}^{-}=>b^{1}\Sigma_{g}^{+}$ transition at λ = 763.4 nm is used. As the main part of the transitional energy of a molecule de-exciting from this energy level is attributed to the electronic transition, a large amount of the photoacoustic signal can be generated by the E-T process.

The exact energy transfer processes in photoacoustic spectroscopy are generally unknown due to the large number of energy levels and molecular species that are involved. For O_2_, some of the transfer rates have been reported for de-excitation in a pure oxygen atmosphere [8–13]. Although these rates neglect per se collisions with background gas molecules, they can give an idea of the formation of the photoacoustic signal. Radiative de-excitation is a loss mechanism for photoacoustics and needs to be taken into account as well.

As a result of the small optical transition probability the fluorescence lifetime of the $b^{1}\Sigma_{g}^{+}=>X^{3}\Sigma_{g}^{-}$ transition is extremely long (τ = 12 s) [8]. This long lifetime favors photoacoustics as it minimizes the energy losses caused by radiative de-excitation. The fluorescence lifetimes for $b^{1}\Sigma_{g}^{+}=>a^{1}\Delta_{g}$ and $a^{1}\Delta_{g}=>b^{1}\Sigma_{g}^{+}$ are even longer [9] and thus de-excitation following this path can be neglected.

Numerical simulations of the energy transfer processes leading to de-excitation of an O_2_ molecule within a pure oxygen atmosphere at room temperature provide de-excitation rates for E-E, E-T, V-V and V-T processes.

For the electronic-electronic transfer process (E-E process), where a collision leads to the electronic excitation of an O_2_ molecule that was previously in the ground state, a removal rate coefficient of Γ = 1.5 × 10^−11^ cm^3^·molecules^−1^·s^−1^ for the first excited vibrational level is derived [10]. For the E-T process the rate coefficient is Γ = 4 × 10^−18^ cm^3^·molecules^−1^·s^−1^ for the same starting energy levels [11].

The V-V transfer rate within the ground state, *i.e.*, the collision with another molecule in the ground state and its excitation into a vibrational mode, is also some orders of magnitude larger than the conversion to translation energy, *i.e.*, the V-T process [12].

Calculations of the lifetime of the excited molecules at *p* = 250 mbar pressure, which is typical for QEPAS measurements, with respect to the rate constants given above, lead to τ = 10.98 ns for the E-E process and τ = 41.16 ms for the E-T process. Therefore the energy transfer to other O_2_ molecules is much more efficient than the E-T relaxation and the lifetime three orders of magnitude larger than the oscillation period of approximately *T* = 30 μs for the tuning fork in the QEPAS experiments. This results in a poor QEPAS performance as only a small fraction of the energy is used for sound wave generation. Also, the transfer rates for collisions with molecules other than O_2_ are small, e.g., N_2_, CO_2_ [13]. For water vapor, which is known to be effective as a collisional de-excitation partner, no values are available in the literature.

### 2.2. Experimental setup

To compensate for the ineffective generation of photoacoustic signals, the optical power was increased with a tapered amplifier to enhance the signal according to Equation (1). A tapered amplifier is a semiconductor device that consists, similar to a diode laser, of an electrically pumped active laser medium. The name is deduced from the geometric form of the active zone. Optical amplifiers are operated most effectively in the regime of gain saturation, *i.e.*, the intensity of the seed laser is chosen high enough to remove the entire population inversion within the active medium. However, this also implies that the output power is not linearly dependent on the seed power, but the gain decreases with increasing power. As discussed later, this has influence on the symmetry properties of the measured 2f-spectra.

**Figure 2.**
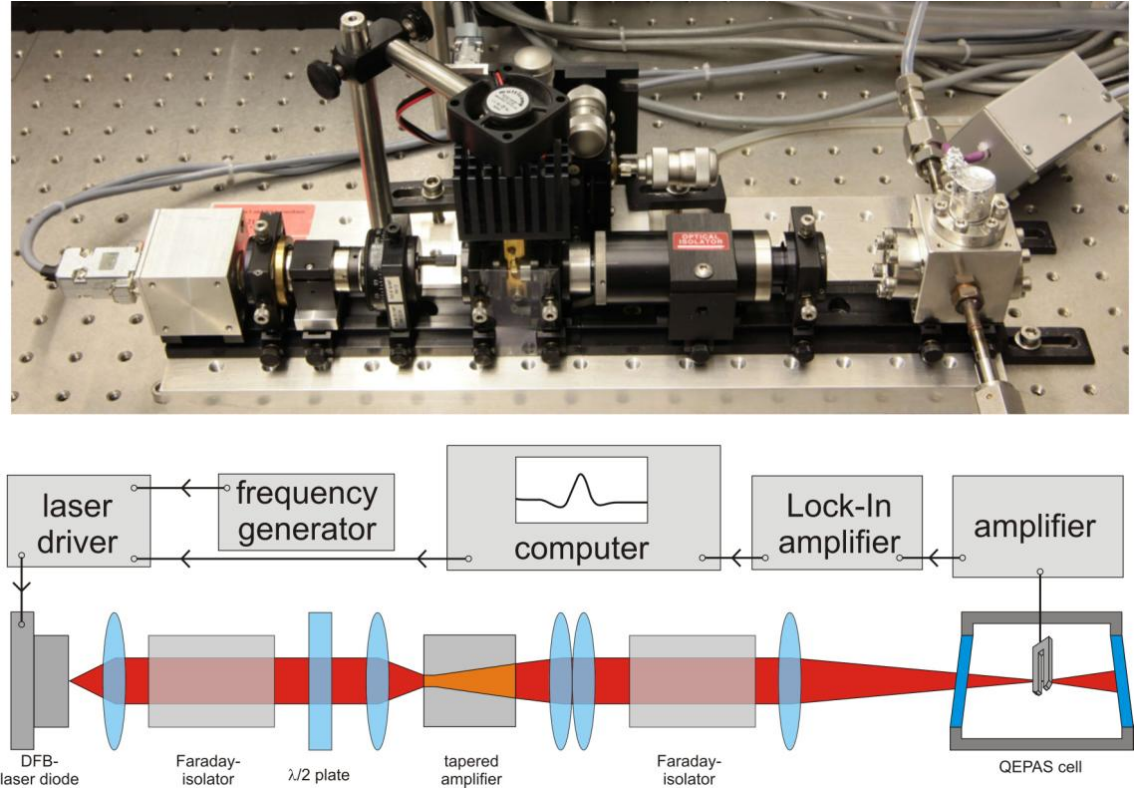
Optical setup with tapered amplifier, electronics and data acquisition. The upper part shows a photograph of the implemented system while the lower part is a schematic drawing of the system.

The experimental setup is shown in Figure 2, showing both a photograph as well as a schematic illustration in the lower part. The sequence of optical elements from left to right is the same for photograph and scheme. The setup can be segmented into blocks to facilitate the description in more detail: the seed laser, the tapered amplifier, the photoacoustic cell, and electronics devices. The seed laser is a distributed feedback (DFB) laser diode (Eagleyard EYP-DFB-0763-00050) operating at λ = 763 nm and with a nominal optical output power of *P* = 50 mW. The center wavelength of the diode is determined by the temperature, while a current ramp is applied to tune the wavelength for spectroscopic measurements. In addition, the wavelength is sinusoidal modulated via the driving current at *f_0_*/2 were *f_0_* is the resonance frequency of the tuning fork for 2f wavelength modulation spectroscopy.

After collimation, the laser beam travels through a Faraday isolator to protect the diode laser from back reflections. A half wave plate is used to adjust the polarization before the beam is focused into the tapered amplifier (Eagelyard EYP-TAP-0765-01500). It is operated at a temperature *T* = 12.5 °C and a current *I* = 2.55 A. Because of the spatial dimensions of the active layer, the beam has an oval profile with dimensions of approx. 0.75 mm by 4 mm (FWHM). The light is re-collimated in both directions by a system of an aspheric (*f* = 4.03 mm) and a cylindrical lens (*f* = 30 mm). A second Faraday-isolator protects the tapered amplifier from reflections. The beam is then focused through the tubes of the acoustic resonator (inner diameter D = 400 μm) and between the prongs of the tuning fork with a spherical lens (*f* = 80 mm). The tapered amplifier has a maximum output power of 1.5 W. Due to losses in the optical elements, there is a maximum power of 1.2 W inside the QEPAS cell. For measurements at low optical powers the DFB laser diode is focused directly into the cell without use of the amplifier.

The acoustic resonator consists of two stainless steel tubes with a length of approximately 5 mm and enhances the signal by a factor of approximately 20. The QEPAS cell is ultra high vacuum sealed to prevent oxygen contamination by leakage and has a volume of approximately *V* = 10 cm^3^. The electronics and data acquisition system consists of a laser driver and function generator for the tuning and modulation of the laser, and an amplifier system. The piezoelectric signal of the tuning fork is amplified by a transimpedance amplifier and the 2f wavelength modulation signal is detected via lock-in technique. A computer controls the individual measurements and stores the data.

## 3. Results

**Figure 3.**
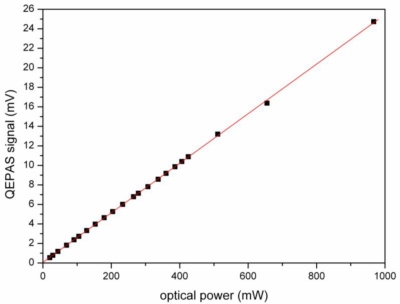
Dependence of the QEPAS signal on the optical power, measured at *C* = 21% oxygen concentration in air. From the linear fit (line) a slope of 25.4 mV/W is derived.

The tapered amplifier has two main effects on the measurement. First, it enhances the photoacoustic signal according to Equation (1), *i.e.*, the QEPAS signal increases linearly with the incident optical power. Figure 3 shows a QEPAS measurement at different optical powers; the linear dependence is clearly visible. Despite the high power of the laser, no offset was experienced which indicates that the alignment through the spectrophone is acceptable.

**Figure 4.**
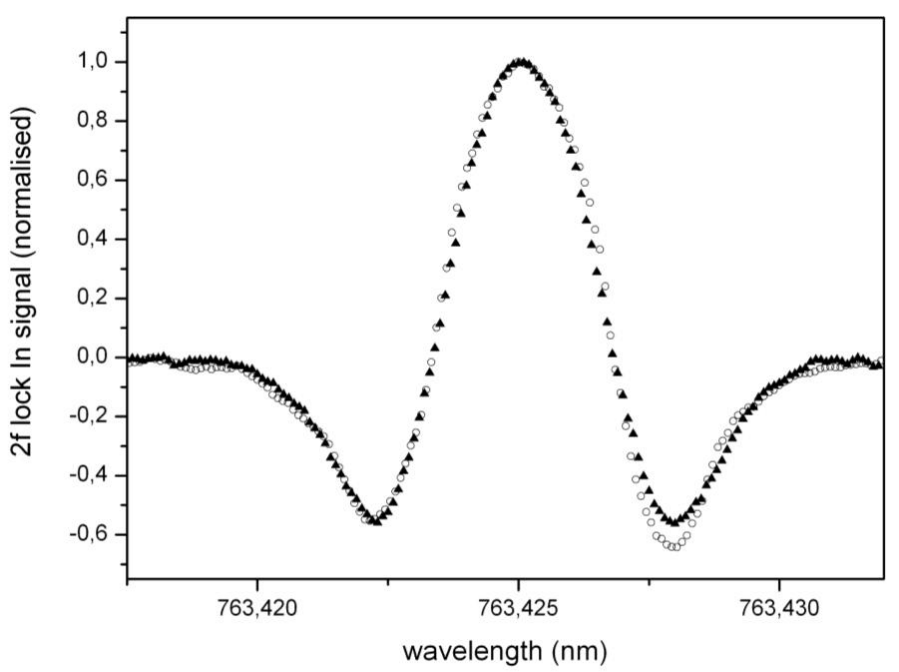
QEPAS measurements of ambient oxygen in air at *p* = 300 mbar. The graph shows the normalized 2f wavelength modulation signals measured without (circles) and with the tapered amplifier (triangles).

Secondly, the tapered amplifier reduces the effect of unwanted amplitude modulation that occurs simultaneously with the wavelength modulation of the DFB diode laser with laser current. The amplitude modulation results in a deformation of the spectral line shape for wavelength modulation spectroscopy [14]. Figure 4 shows this effect by comparing the signals measured without and with the amplifier.

In theory, the 2f signal is proportional to the second derivative of the line shape function and thus should be symmetrical. However, the measurement without the amplifier shows a slight asymmetry that is a result of the wavelength dependent intensity. Figure 4 also shows a measurement using the amplifier; this measurement shows asymmetry to a much lesser extent. This is the result of the nonlinear amplification factor of the tapered amplifier that is operated well above the small signal amplification. Therefore, the output of the tapered amplifier is less sensitive to changes of the seed power and the relative change in optical power, while tuning the wavelength of the DFB laser will be reduced. The average power of the DFB laser diode increases by 23.5% over a wavelength change of Δλ = 14.4 pm. If the amplified system is used, the power increase is only 3.5%. The frequency modulation optimized for 2f-spectroscopy at atmospheric pressure is associated with an amplitude modulation of 0.55% with the DFB diode; this is reduced to 0.004% when the tapered amplifier is used. Thus, the amplifier strongly reduces line shape distortion as shown in Figure 4.

**Figure 5.**
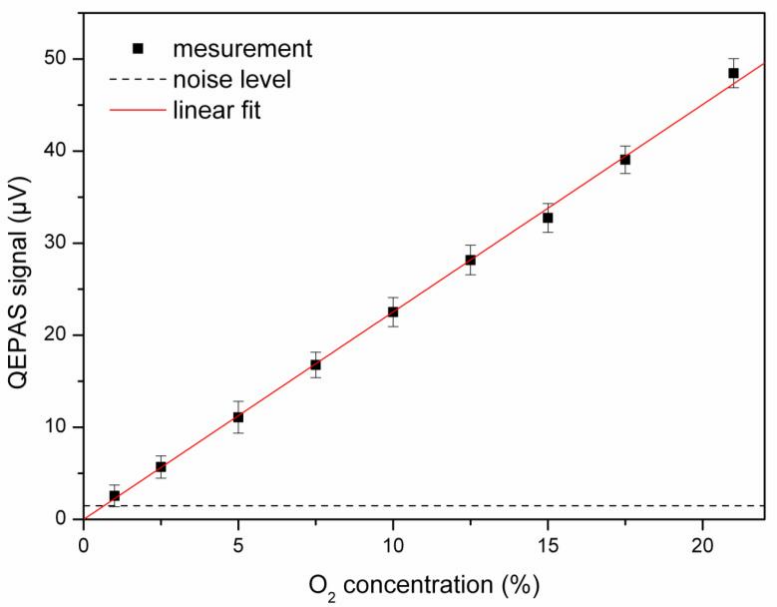
Calibration of the sensor without tapered amplifier. From the numerical fit, a coefficient of 4.44·10^5^ %/V is determined. The noise level of 1.48 μV is shown as a broken line.

Calibration and determination of the detection limit were done for both systems, *i.e.*, with and without the tapered amplifier. In both cases argon (Westfalen, purity 99.996%) taken from a cylinder is used as background gas. For the measurement without the tapered amplifier, the oxygen concentration is regulated by a calibrated gas mixing system consisting of mass flow controllers. The mixed gas is then directed through the QEPAS cell. Figure 5 shows the QEPAS signal as a function of oxygen concentration for the DFB laser diode as the light source. The error bars result from the standard deviation of 100 consecutive measurements. The measurement is done with a lock-in amplifier time constant of τ = 1 s with a roll off of 24 dB/oct and at an absolute pressure of *p* = 250 mbar inside the cell. This was found to be the pressure for which the best signal was obtained in this setup. The average optical power at the center wavelength of the absorption line was *P* = 25 mW. From the slope, a calibration factor for calculating the concentration *C* from the measured QEPAS signal *S* can be derived: *C* = 4.44·10^5^ %/V·*S*. Considering the average of the individual standard deviations as noise level (1.48 μV) one finds a detection limit of 0.66% (1 σ) for oxygen.

For the measurement with the tapered amplifier, smaller concentrations have to be set; therefore an enclosed gas circulating system was used. The oxygen partial pressure in this ultrahigh vacuum sealed system is set by an oxygen ion pump and a λ-probe is used as a reference oxygen sensor while the gas flow is maintained by a circulating pump.

**Figure 6.**
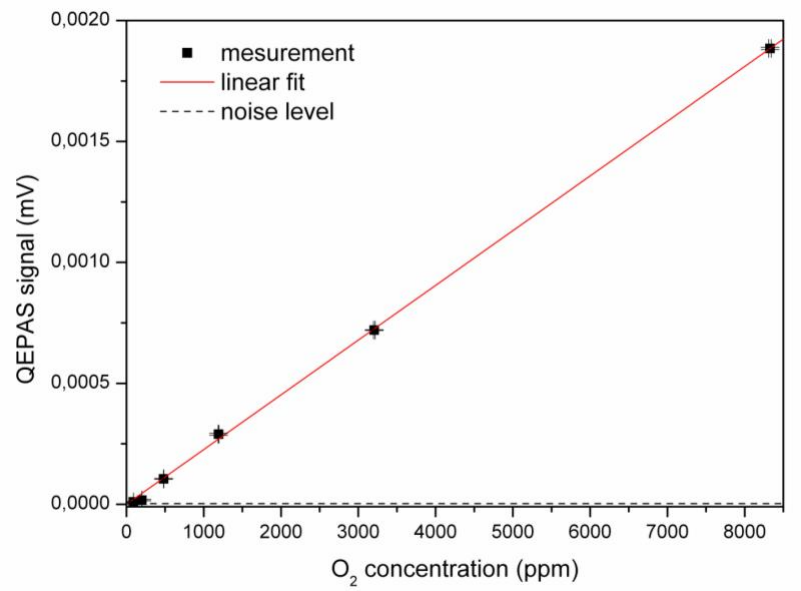
Calibration of the detector with tapered amplifier.

The dependence of the QEPAS signal on the oxygen concentration for measurements in this setup is shown in Figure 6. The points result from averaging 50 consecutive measurements, the error bars are the standard deviations. The pressure within the system was set to *p* = 210 mbar for technical reasons and deviates thus slightly from the optimum value for QEPAS. The lock-in amplifier settings were the same as those used for the experiments without the tapered amplifier (τ = 1 s, roll off 24 dB/oct). In this case, a detection limit of 13 ppm was found for oxygen (1 σ). The corresponding coefficient was 4.41·10^6^ ppm/V and the noise level was determined to be 2.93 μV. These data were obtained at an optical power of *P* = 1228 mW. Accounting for the laser power used for excitation and the bandwidth of the detection system, this value leads to a power and bandwidth normalized noise equivalent absorption sensitivity (NEAS) of *D* = 4.74 × 10^−7^ cm^−1^ W/Hz^1/2^.

**Figure 7.**
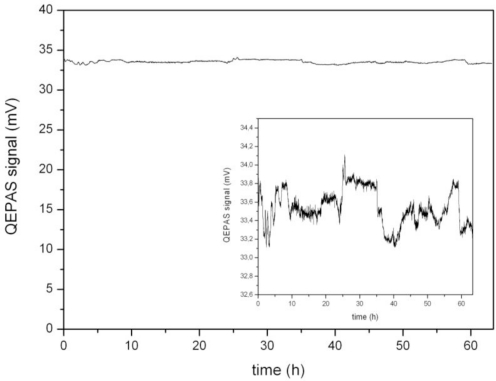
Long-term QEPAS measurement of oxygen in air with the amplified laser system. The signal is normalized to an optical power of *P* = 1 W. The signal variation is found to be 0.57% (RMS). The inset shows the same measurement with another scale.

The dominant noise source for resonant measurements with quartz tuning forks is the thermal or Johnston noise of the tuning fork [15]. The noise caused by the tuning fork is therefore independent of the signal strength and the resolution for measurements of higher concentrations is determined by the noise floor, which also defines the detection limit. As the measurements with and without the tapered amplifier were done in different setups with two different spectrophones (consisting of tuning fork and acoustic resonator) the measurements are not directly comparable as the Q-factor of the tuning fork and the acoustic resonator both influence the signal amplitude and the noise level. The long-term stability of the system is tested by performing a measurement of the atmospheric oxygen concentration in an enclosed cell over more than 60 hours (Figure 7).

During the measurement, laser wavelength and resonance frequency were monitored and the modulation frequency and laser current were adjusted to maintain operation at the maximum signal. The optical power incident into the cell is also recorded using a reflection from the wedged input window. It turned out that the change of resonance frequency is less than 0.1 Hz and thus negligible since the resonance curve width is approximately 30 Hz (FWHM). Therefore, in principle, it is not necessary to lock the resonance frequency as long as no change in pressure occurs. The relatively low wavelength drift of Δλ = 0.4 pm peak to peak was compensated for by readjustment of the laser current. A variation in the output power of the amplified laser [*ΔP* = 1.57% (RMS)], however, showed much influence on the measured signal, therefore the monitored power was used to normalize the signal to an optical power of *P* = 1 W. The result is shown in Figure 7. The remaining fluctuations, which can be assessed in more detail in the inset which shows the same data with a different scale, are attributed mainly to temperature and mechanical instabilities of the optical coupling into the tapered amplifier. These effects are difficult to suppress as the adjustment of a relative heavy load (amplifier including temperature stabilization equipment) in the sub-micron range is difficult to maintain over long periods of time. Normalizing the QEPAS signal to the optical power reduces the signal variation to 0.57% (RMS) of the measured concentration, which determines the long-term accuracy of the concentration measurements. Depending on the accuracy needed for a special application, the time interval for automated re-calibrations has to be adapted. Multiple measurements with different spectrophones have been performed and show comparable results. It has to be pointed out however, that calibration needs to be performed for the single device and is not transferable between systems.

**Figure 8.**
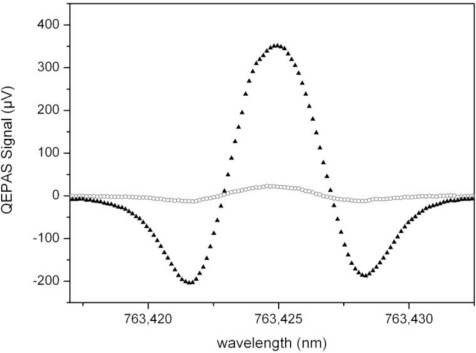
2f QEPAS signals of 21% oxygen in dry argon (circles) and ambient air (triangles). The contained water vapor leads to a signal enhancement due to higher V-T transfer rates.

Another factor influencing the measurement in air is humidity. Figure 8 shows a comparison of a measurement with 21% oxygen in dry argon as background gas taken from a cylinder and another one under the same conditions, but in ambient air with 35.8% relative humidity at 24.6 °C. The signal in air is enhanced by a factor of 15.6.

The reasons for these results are the large E-E and V-V transition rates of oxygen. As water is known for its efficient V-T relaxation [16,17], the PAS signal is much enhanced when the energy from oxygen is transferred to water were the V-T process produces a strong signal due to higher transfer rates. Therefore, further investigations are needed to optimize the gas composition by adding small amounts of a third gas, e.g., water or SF_6_, which is also known to give rise to a photoacoustic signal enhancement.

## 4. Conclusions

Despite the unfavorable photoacoustic properties of oxygen, the applicability of the QEPAS technique for the detection of low oxygen concentrations with a high power amplified laser system has been demonstrated. The high optical power does not influence the noise floor or the linearity of the signal, but reduces signal distortions due to a reduction of unwanted amplitude modulation. With the system, a detection limit of 13 ppm is reached with a compact and long term stable setup. As the signal strength shows a strong dependence to additional components of the gas mixture, a detection limit below 1 ppm should be possible with an optimized gas mixture.
